# The Effect of a Novel Serine Protease Inhibitor on Inflammation and Intestinal Permeability in a Murine Colitis Transfer Model

**DOI:** 10.3389/fphar.2021.682065

**Published:** 2021-06-24

**Authors:** Hanne Van Spaendonk, Hannah Ceuleers, Annemieke Smet, Maya Berg, Jurgen Joossens, Pieter Van der Veken, Sven M. Francque, Anne-Marie Lambeir, Joris G. De Man, Ingrid De Meester, Koen Augustyns, Benedicte Y. De Winter

**Affiliations:** ^1^ Laboratory of Experimental Medicine and Pediatrics, University of Antwerp, Antwerp, Belgium; ^2^ Infla-Med, Centre of Excellence, University of Antwerp, Antwerp, Belgium; ^3^ Laboratory of Medicinal Chemistry, University of Antwerp, Antwerp, Belgium; ^4^ Division of Gastroenterology and Hepatology, Antwerp University Hospital, Antwerp, Belgium; ^5^ Laboratory of Medical Biochemistry, University of Antwerp, Antwerp, Belgium

**Keywords:** T cell transfer colitis mouse model, serine protease inhibitor, intestinal barrier, T cells, protease-activated receptors

## Abstract

**Background:** A protease/antiprotease disbalance is observed in inflammatory bowel diseases (IBD). We therefore studied the effect of the novel serine protease inhibitor UAMC-00050 on intestinal inflammation and permeability in a chronic colitis T cell transfer mouse model to get further insight into the regulation of T cell-mediated immunopathology.

**Methods:** Colitis was induced in severe combined immunodeficient (SCID) mice, by the adoptive transfer of CD4^+^CD25^−^CD62L^+^ T cells. Animals were treated intraperitoneally (i.p.) 2x/day with vehicle or UAMC-00050 (5 mg/kg) from week 2 onwards. Colonic inflammation was assessed by clinical parameters, colonoscopy, macroscopy, microscopy, myeloperoxidase activity and cytokine expression levels. At week 4, 4 kDa FITC-dextran intestinal permeability was evaluated and T helper transcription factors, protease-activated receptors and junctional proteins were quantified by RT-qPCR.

**Results:** Adoptive transfer of CD4^+^CD25^−^CD62L^+^ T cells resulted in colonic inflammation and an altered intestinal permeability. The serine protease inhibitor UAMC-00050 ameliorated both the inflammatory parameters and the intestinal barrier function. Furthermore, a decrease in colonic mRNA expression of Tbet and PAR4 was observed in colitis mice after UAMC-00050 treatment.

**Conclusion:** The beneficial effect of UAMC-00050 on inflammation was apparent via a reduction of Tbet, IFN-γ, TNF-α, IL-1β and IL-6. Based on these results, we hypothesize a pivotal effect of serine protease inhibition on the Th1 inflammatory profile potentially mediated via PAR4.

## Introduction

Inflammatory bowel diseases (IBD) are chronic inflammatory diseases of the gastrointestinal tract and include ulcerative colitis (UC) and Crohn’s disease (CD). The etiology of IBD is still not fully elucidated, but it involves a complex interaction between environmental, host genetic, epithelial and microbiome-derived factors next to an uncontrolled immune reaction (Chang, 2020). Recently, there is a renewed interest in the role of the intestinal barrier in the pathophysiology of gastrointestinal diseases as genetic profiles, microbiota dysbiosis and inflammatory mediators all have the potential to damage the barrier integrity, hereby increasing tissue destruction and mucosal inflammation even more (Odenwald and Turner, 2017; Mehandru and Colombel, 2020; Hanning et al., 2021a).

Next to inflammatory mediators such as the cytokines TNF-α, IL-6 and IL-1β, also proteases have been put forward to play a pivotal role in the regulation of the intestinal barrier function (Sola Tapias et al., 2020). Proteases are enzymes that are expressed throughout the entire body where they regulate a variety of biological processes such as coagulation, cell growth and migration, apoptosis and protein catabolism. In the gastrointestinal tract, high concentrations of proteases are present playing a well-known role in digestive processes but also contributing to inflammation, visceral hypersensitivity and permeability disturbances (Ceuleers et al., 2016; Vergnolle, 2016; Van Spaendonk et al., 2017). In the intestinal context, proteases are produced by the pancreas, immune cells (e.g., macrophages, neutrophils, mast cells, dendritic cells), but also by intestinal epithelial cells and specific microbial strains (Carroll and Maharshak, 2013; Vergnolle, 2016). On the one hand, proteases act as signaling molecules via specific PARs, on the other hand, they degrade mucosal proteins (e.g. mucins) (Paone and Cani, 2020) and extracellular matrix. In addition, cytokines and chemokines can be cleaved by proteases modulating their bioactivity (Vergnolle, 2016; Van Spaendonk et al., 2017). On their turn, the bioactivity of proteases is regulated by endogenous protease inhibitors such as serpins, chelonianins and tissue inhibitors of metalloproteinases (TIMPS) (Vergnolle, 2016). A disbalance between proteases and protease inhibitors is, however, observed in gastrointestinal diseases such as IBD. Indeed, intestinal tissue from IBD patients shows an upregulation at the mRNA, protein and/or activity level of serine protease (e.g. cathepsin G, tryptase, thrombin, plasma kallikrein, chymotrypsin-like elastase 3A, plasmin and aminopeptidase B), cysteine protease, aspartate protease and metalloprotease families (Cleynen et al., 2011; Vergnolle, 2016; Denadai-Souza et al., 2018; Sola Tapias et al., 2020). The findings on the expression of the protease inhibitors are, however, more diverse compared to the expression levels of proteases, reporting increases, decreases or no changes in expression. This highlights the importance of investigating the biological activity of proteases next to solely the expression levels. Moreover, very recently Motta *et al.* showed the role of active thrombin, produced by epithelial cells, in biofilm formation at the level of the intestinal mucosa (Motta et al., 2019).

A method to influence the protease signaling pathways is to directly alter protease activity by using selective inhibitors (Vergnolle, 2016). In this respect, we previously developed selective serine protease inhibitors (patents WO/2007/045496, WO/2017/198753) (Joossens et al., 2007) and recently showed their *in vivo* therapeutic potential in inflammation-induced visceral hypersensitivity (Ceuleers et al., 2018; Hanning et al., 2021b) and dry eye derived ocular inflammation (Joossen et al., 2020). Besides, UAMC-00050 displays a favorable inhibitory profile with a good inhibition of the serine protease tryptase, together with a limited inhibition of proteases involved in the coagulation cascade (Ceuleers et al., 2018). Furthermore, we recently demonstrated that the pharmacokinetic properties of UAMC-00050 are suitable for translation to a clinical setting, since the compound is only minimally detected in blood samples after local colonic administration (Hanning et al., 2021b). These promising results formed the basis of this study aiming at investigating the effect of the trypsin-like serine protease inhibitor UAMC-00050 on intestinal permeability and intestinal inflammation in a mouse model of chronic colitis.

## Materials and Methods

### Mice

Severe Combined Immunodeficient (SCID) (CB17/Icr-Prkdcscid/IcrIcoCrl) and BALB/c mice were obtained from Charles River (France). All animals were female, 8–9 weeks old at the initiation of the experiment and housed in a conventional animal facility with ad libitum access to food and water. All experiments were approved by the Ethical Committee on animal experimentation of the University of Antwerp (file number 2014-42).

### T Cell Transfer Model of Chronic Colitis

Chronic colitis was induced by the adoptive transfer of splenic CD4^+^CD25^−^CD62L^+^ T cells into immunocompromised recipient mice as previously described (Heylen et al., 2013). Previously published work from our research group (Breugelmans et al., 2020) demonstrated that both the DSS colitis mouse model as well as the adoptive T cell transfer mouse model are adequate models to study intestinal inflammation and barrier dysfunction. For these experiments, we opted for the adoptive T cell transfer model, which is of main interest when studying immunological mechanisms of intestinal inflammation mediated by T cells. Moreover, our research group previously demonstrated the therapeutic potential of helminth-derived products in this experimental animal model of colitis (Heylen et al., 2014; Heylen et al., 2015). In brief, a single cell suspension was prepared from the spleens of donor BALB/c mice, from which CD4^+^CD25^−^CD62L^+^ T cells were isolated using a magnetic CD4^+^CD62L^+^ T cell isolation kit (Miltenyi Biotec GmhB). To induce colitis, SCID mice were injected i.p. with 1 × 106 CD4^+^CD25^−^CD62L^+^ T cells in 100 µl phosphate-buffered saline (PBS). Control BALB/c mice were i.p. injected with PBS and remained healthy.

### Experimental Design

**FIGURE 1:**
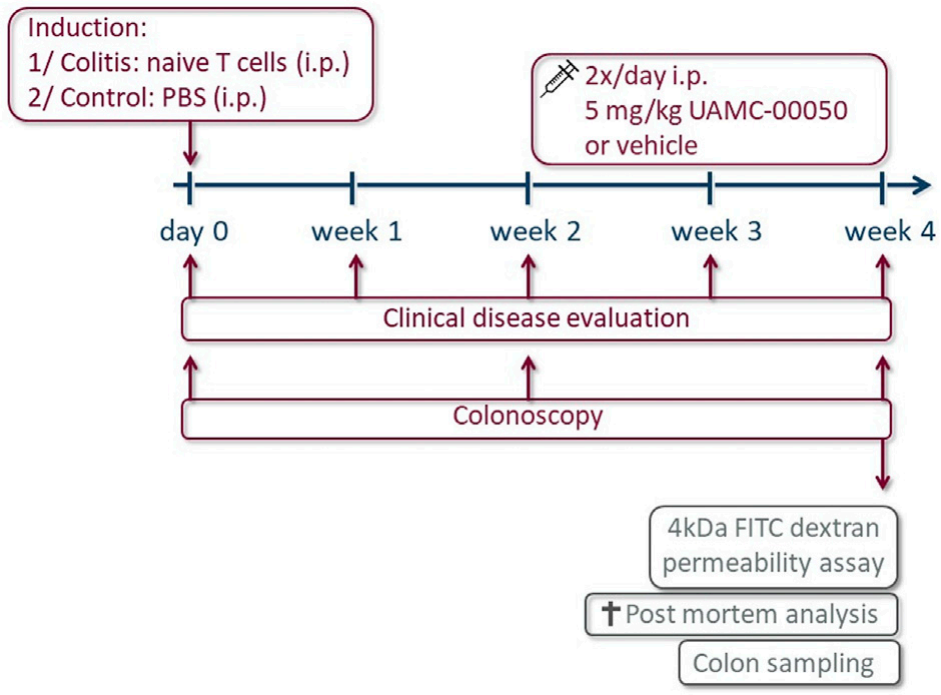
Overview of the experimental design. On day 0, mice received an intraperitoneal (i.p.) administration with naive T cells (colitis) or PBS (control). The clinical disease score was evaluated every week and a colonoscopy was performed every other week for 4 weeks. From week 2 onwards, animals were injected i.p. with the serine protease inhibitor UAMC-00050 or vehicle 2x/d in a dose of 5 mg/kg. Final experiments were conducted after 4 weeks: intestinal permeability was determined by 4 kDa FITC-dextran and after sacrifice, a post-mortem analysis was executed to evaluate the inflammatory parameters and colon sampling was performed for the determination of cytokines via CBA and transcription factors, PARs and tight junctions via qPCR.

The experimental design is shown in Figure 1. After induction of colitis by T cell transfer, the gradual development of colitis was evaluated using a clinical disease score every week for 4 weeks and by colonoscopy every second week. Four weeks after induction of colitis, mice were gavaged with 4 kDa FITC-dextran to determine intestinal permeability and sacrificed for post-mortem analysis, prelevating colonic tissue for macroscopic evaluation, molecular biology of transcription factors, junctional proteins and PARs, CBA determination of the cytokines and MPO activity. Blood was drawn by cardiac puncture before removing the colon, for the assessment of the concentration of 4 kDa FITC-dextran in serum by fluorospectrometry.

Control and colitis mice were treated with UAMC-00050 in a dose of 10 mg/kg/day, administered as two i.p. injections of 5 mg/kg/day, or the vehicle (5% DMSO solution in PBS) starting 2 weeks after T cell transfer. Each group consisted of *n* = 7–8 mice.

UAMC-00050 (benzyl (1-(bis(4-acetamidophenoxy)phosphoryl)-2-(4-guanidinophenyl)ethyl)carbamate hydrochloride) is an inhibitor of trypsin-like serine proteases with a well-defined multi-target inhibition profile (Joossens et al., 2007; van Soom et al., 2015; Ceuleers et al., 2018). This compound was developed by the Laboratory of Medicinal Chemistry of the University of Antwerp, originally patented under WO2007045496 and later on also patented for its use in PAR-related diseases (WO2017198753).

### Clinical Examination

To follow up disease progression, mice were weighed every week and scored as previously reported (Heylen et al., 2013; Breugelmans et al., 2020) based on the following clinical disease parameters: weight loss, piloerection, stool consistency and mobility. Each parameter was graded from 0 to 2 according to disease severity (0 = absent, 1 = moderate, 2 = severe; for weight loss, 0 = weight gain, 1 = stable, 2 = weight loss). The cumulative score reached from 0 to 8.

### Colonoscopic Examination of the Colon

To monitor intestinal inflammation in a continuous manner in individual mice, colonoscopy was performed at fixed time points (weeks 0, 2, and 4) as previously described using a flexible Olympus URF type P5 ureteroscope with an outer diameter of 3.0 mm (Olympus Europe GmbH) (Heylen et al., 2013). Briefly, mice were sedated with a mixture of ketamine (60 mg/kg, Ketalar®, Pfizer) and xylazine (6.67 mg/kg, Rompun®, Bayer) (i.p.) and placed in prone position. The anal sphincter was lubricated with gel (RMS-endoscopy) to facilitate insertion of the endoscope. Subsequently, the scope was carefully inserted through the anus as far as possible into the descending colon of the sedated mouse. A score was given during the withdrawal of the scope for the following parameters: morphology of the vascular pattern, bowel wall translucency, fibrin attachment and the presence of loose stools (each ranging from 0 to 3), with a cumulative minimum of 0 (no inflammation) and a maximum of 12 (severe inflammation).

### Macroscopic Inflammation Score

Upon termination of the experiment (week 4), mice were sacrificed by exsanguination under anesthesia (90 mg/kg ketamine and 10 mg/kg xylazine) and the colons were removed as described previously (Heylen et al., 2013). Feces were removed and the weight and length of the colon were determined and expressed as weight/length ratio (mg/cm). Subsequently, the colon was scored for macroscopic signs of inflammation based on the following parameters: presence of ulcerations, hyperemia, bowel wall thickening and mucosal edema. Each parameter was scored from 0 to 3 depending on the severity, leading to a maximum cumulative score of 12 (Heylen et al., 2013).

### Microscopic Inflammation Score

Full thickness colonic segments, taken in a standardized way, were fixed for 24 h in 4% formaldehyde and subsequently embedded in paraffin. Cross sections (5 µm) were stained with hematoxylin-eosin. Inflammation was scored in a blinded fashion based on the degree of inflammatory infiltrates (0–3), presence of goblet cells (0–1), crypt architecture (0–3), mucosal erosion and/or ulceration (0–2), presence of crypt abscesses (0–1) and the number of layers affected (0–3), resulting in a cumulative score ranging from 0 to 13 (Heylen et al., 2013).

### MPO Activity Assay

Myeloperoxidase (MPO) activity was measured in colonic tissue as a parameter for tissue inflammation. The MPO activity directly correlates to the number of active neutrophil granulocytes as MPO is stored in the granules within the neutrophils and released upon degranulation. The assay was performed as described previously (Moreels et al., 2004; Ruyssers et al., 2009; Heylen et al., 2013). Briefly, colonic samples were taken in a standardized way, blotted dry, weighed and placed in a potassium phosphate buffer (pH = 6.0) containing 0.5% hexadecyltrimethylammoniumbromide at a ratio of 100 ml per 5 g tissue. Samples were homogenized and subjected to two freeze-thawing cycles. Subsequently, samples were centrifuged at 15,000 rpm for 15 min at 4°C. An aliquot (0.1 ml) of the supernatant was added to 2.9 ml of o-dianisidine solution (16.7 mg of o-dianisidine dihydrochloride in 1 ml of methyl alcohol, 98 ml of 50 mM potassium phosphate buffer at pH 6.0 and 1 ml of 0.005% H_2_O_2_ solution). The change in absorbance of the samples was read at 460 nm over 60 s using a Spectronic Genesys 5 spectrophotometer (Milton Roy). One unit of MPO activity equals the amount of enzyme able to convert 1 mmol of H_2_O_2_ to H_2_O per minute at 25°C.

### mRNA Isolation and Quantitative Real-Time Polymerase Chain Reaction

Total RNA from colonic tissue stored in RNA-later, was extracted using the NucleoSpin® RNA plus kit (Macherey-Nagel) following the manufacturer’s instructions. The concentration and quality of the RNA were evaluated using the NanoDrop® ND-1000 UV-Vis Spectrophotometer (Thermo Fisher Scientific). Subsequently, 1 µg RNA was converted to cDNA by reverse transcription using the SensiFast™ cDNA synthesis kit (Bioline). Relative gene expression was then determined by SYBR Green RT-qPCR using the GoTaq qPCR master mix (Promega) on a QuantStudio 3 Real-Time PCR instrument (Thermo Fisher Scientific). Primer sequences are shown in Supplementary Table S1. All RT-qPCR reactions were performed in duplicate and involved an initial DNA polymerase activation step for 2 min at 95°C, followed by 40 cycles of denaturation at 95°C for 15 s and annealing/extension for 1 min at 60°C. Analysis and quality control were performed using Qbase+ software (Biogazelle). Relative expression of the target genes was normalized to the expression of the housekeeping genes GAPDH, RPS29 and RPL4.

### Cytometric Bead Array

To determine colonic cytokines on protein level, colonic segments were taken in a standardized manner, rinsed with PBS, blotted dry and weighed, as previously described (Nullens et al., 2018). The samples were stored on ice in a Tris-EDTA buffer [PBS containing 10 mM Tris, 1 mM EDTA, 0.5% v/v Tween20 containing a protease-inhibitor cocktail (Sigma-Aldrich)] at a ratio of 100 mg tissue per mL buffer. Samples were homogenized and centrifuged (11,000 rpm, 10 min, 4°C), supernatant was stored at −80°C until further analysis.

Colonic cytokine levels were quantified using the CBA Mouse Cytokine Kit (BD Biosciences) for TNF-α, IFN-γ, IL-1β, IL-6, IL-10, IL-17 and IL-23 according to the manufacturer’s instructions. For detection, the BD Accuri C6 flow cytometer was used with FCAP Array software for analysis of the output.

### FITC-Dextran Assay for Intestinal Permeability in Mice

To assess *in vivo* intestinal permeability, mice were gavaged with FITC-dextran (44 mg/100 g body weight, 4 kDa, Sigma) 4 h prior to euthanasia (Gupta and Nebreda, 2014). Mice were anesthetized with a mixture of ketamine (90 mg/kg, Ketalar, Pfizer) and xylazine (10 mg/kg, Rompun, Bayer) (i.p.). Blood was collected via cardiac puncture and transferred into SSTII Advance Blood Collection Tubes (BD Vacutainer). After centrifugation (10,000 rpm, 5 min), serum was collected and diluted with an equal volume of PBS. Aliquots of 100 µl were transferred into a 96-well microplate, each sample in duplo. The concentration FITC in the serum was detected by fluorometry (Fluoroskan Microplate Fluorometer, Thermo Fisher Scientific) with an excitation wavelength of 480 nm and an emission wavelength of 530 nm. The exact FITC concentration in each well was calculated using a serially diluted FITC-dextran solution as a standard curve (0–8,000 ng/ml).

### Statistical Analysis

All data are presented as mean ± SEM. The statistical analysis was performed using SPSS (version 24.0, IBM). Body weight, clinical disease score and colonoscopic score data were analyzed using a Generalized Estimating Equations (GEE) test with Least Significant Difference (LSD) post-hoc test. Post-mortem inflammatory parameters, CBA, qPCR and intestinal permeability data were analyzed with Two-Way ANOVA followed by One-Way ANOVA and LSD post-hoc test when appropriate. A *p*-value < 0.05 was considered statistically significant. Representative graphs were made using GraphPad Prism 7.0.

## Results

### Characterization of Inflammation and Intestinal Permeability Parameters in a T Cell Transfer Colitis Model

**FIGURE 2:**
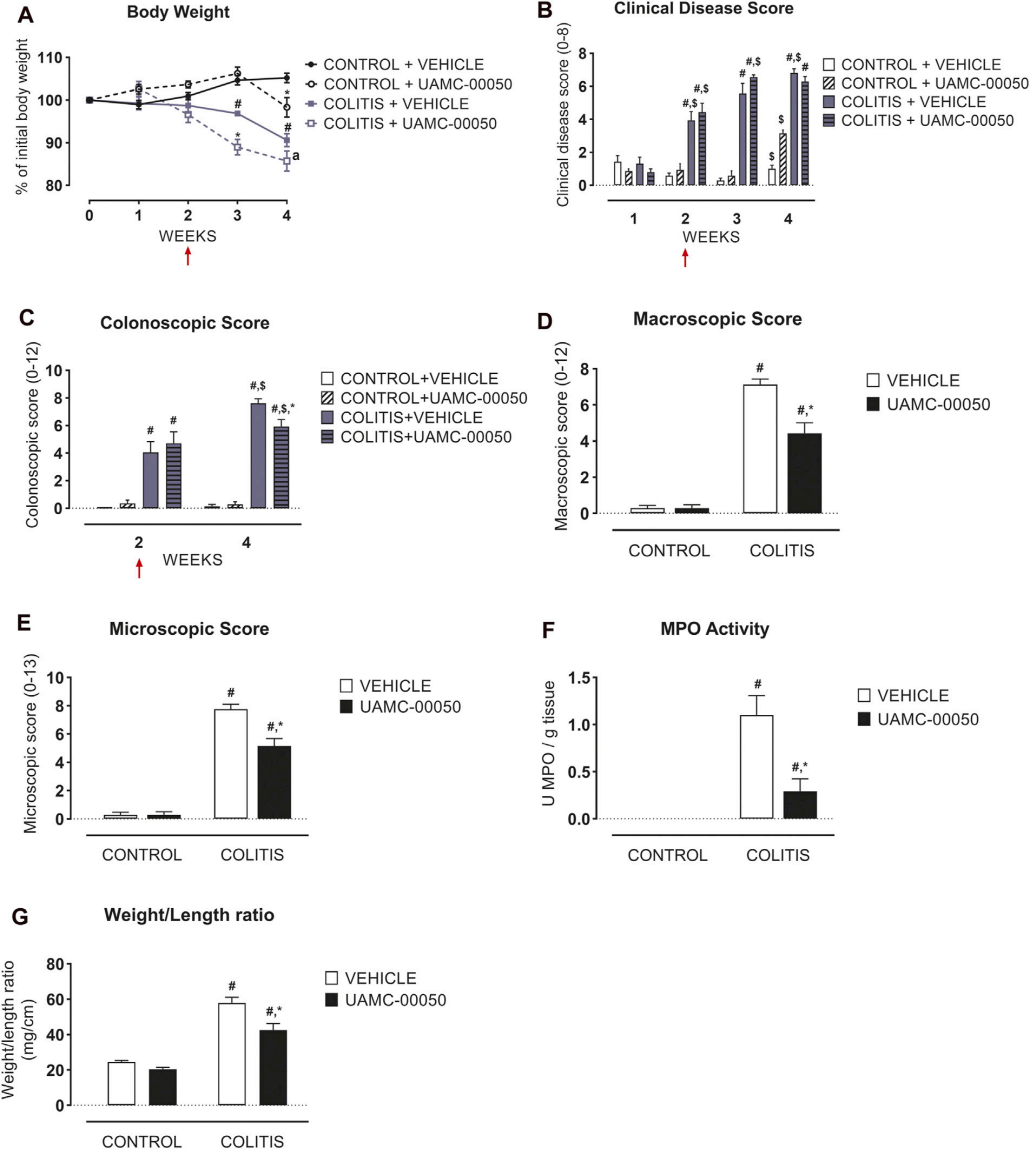
Effect of curative treatment with UAMC-00050 on inflammatory parameters in experimental colitis. Effect on body weight **(A)**, clinical disease score **(B)**, colonoscopic score **(C)**, macroscopic score **(D)**, microscopic score **(E)**, MPO activity **(F)** and colonic weight/length ratio **(G)**. The red arrows indicate the start of the UAMC-00050 treatment in **(A**–**C)**. The data for **(D**–**G)** are post-mortem markers determined at week 4. Data are represented as mean ± SEM. Generalized Estimating Equations (GEE) + Least significant difference (LSD) posthoc test **(A**–**C)**, Two-way ANOVA followed by One-way ANOVA and LSD post-hoc test when appropriate **(D**–**G)**. #*p* < 0.05; significant effect of colitis, *p < 0.05; significant effect of treatment, $ *p* < 0.05; significant effect of time, a *p* = 0.06. *n* = 7–8/group.

**FIGURE 3:**
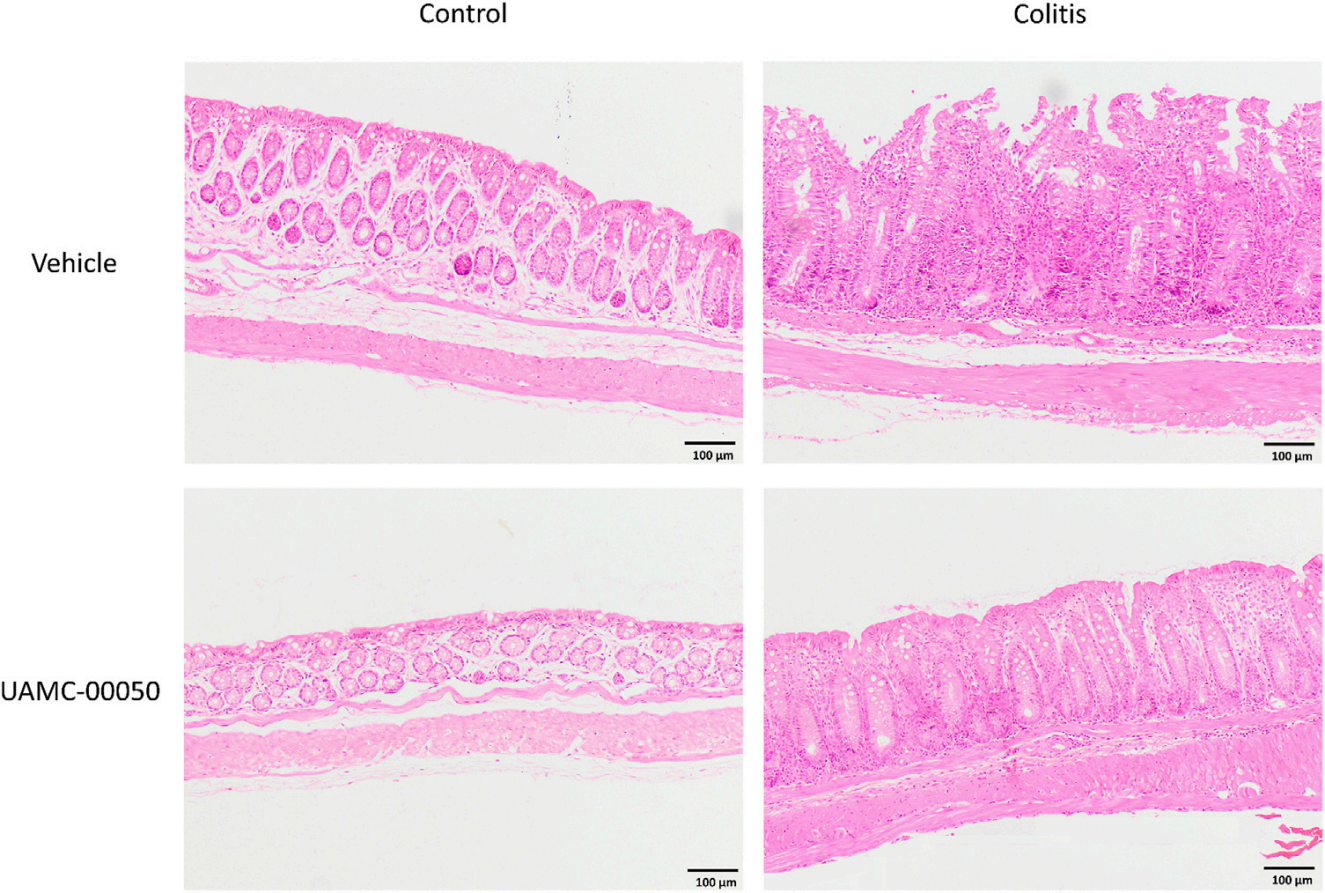
Effect of curative treatment with UAMC-00050 on H&E stained colon sections in experimental colitis. Representative images of H&E stained colon sections (objective × 10, scale bar = 100 µm) from the four different experimental groups (control + vehicle, control + UAMC-00050, colitis + vehicle, colitis + UAMC-00050) that were microscopically scored in Figure 2E.

**FIGURE 4:**
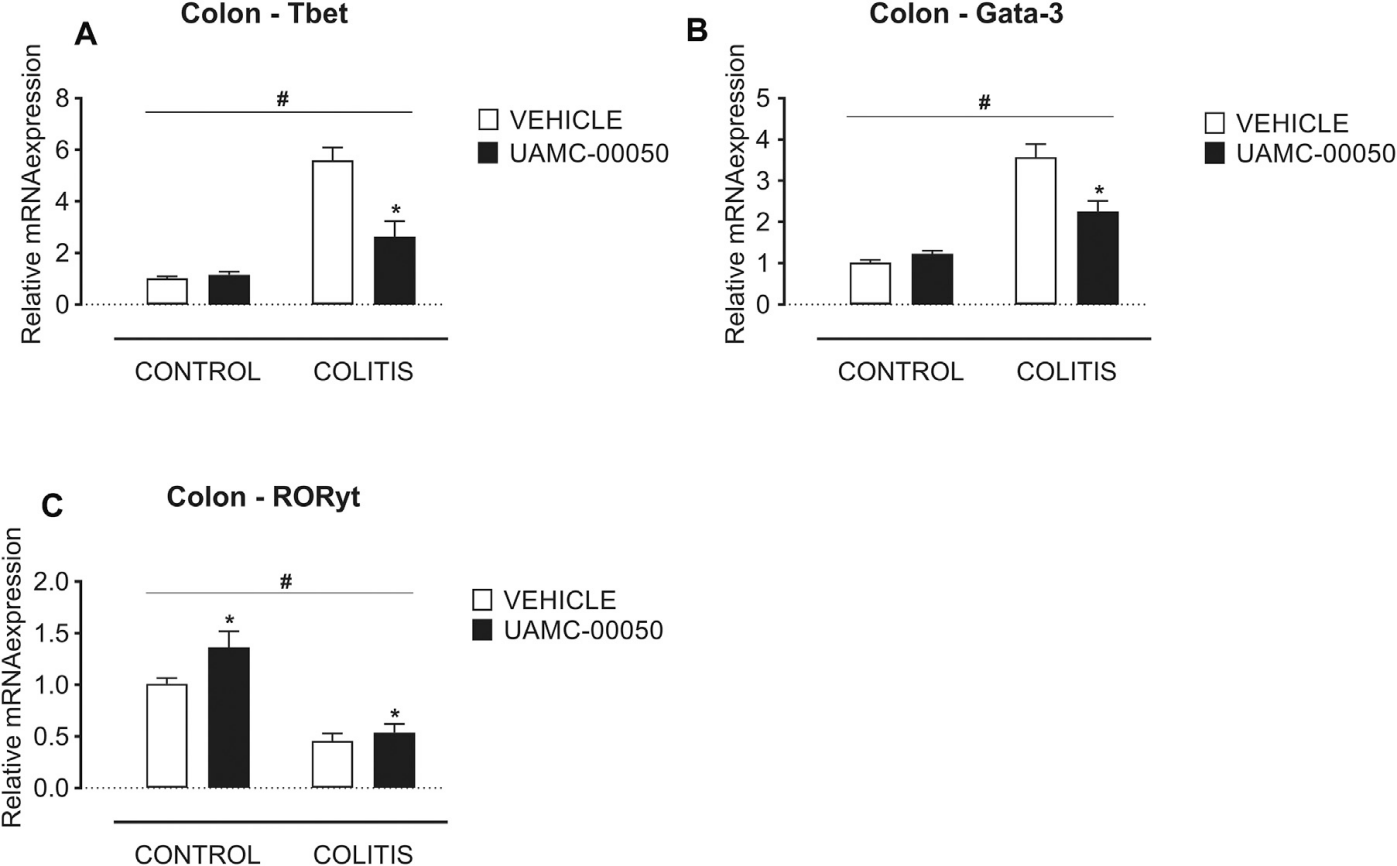
Effect of curative treatment with UAMC-00050 on colonic mRNA expression of Tbet, Gata-3 and RORγt. Effect on expression of Tbet **(A)**, Gata-3 **(B)** and RORγt **(C)**, determined post-mortem at week 4 (after a 2-week treatment with UAMC-00050). Data are represented as mean ± SEM. Two-way ANOVA followed by One-way ANOVA and LSD post-hoc test when appropriate. #*p* < 0.05; significant effect of colitis, *p < 0.05; significant effect of treatment, *n* = 7–8/group.

**FIGURE 5:**
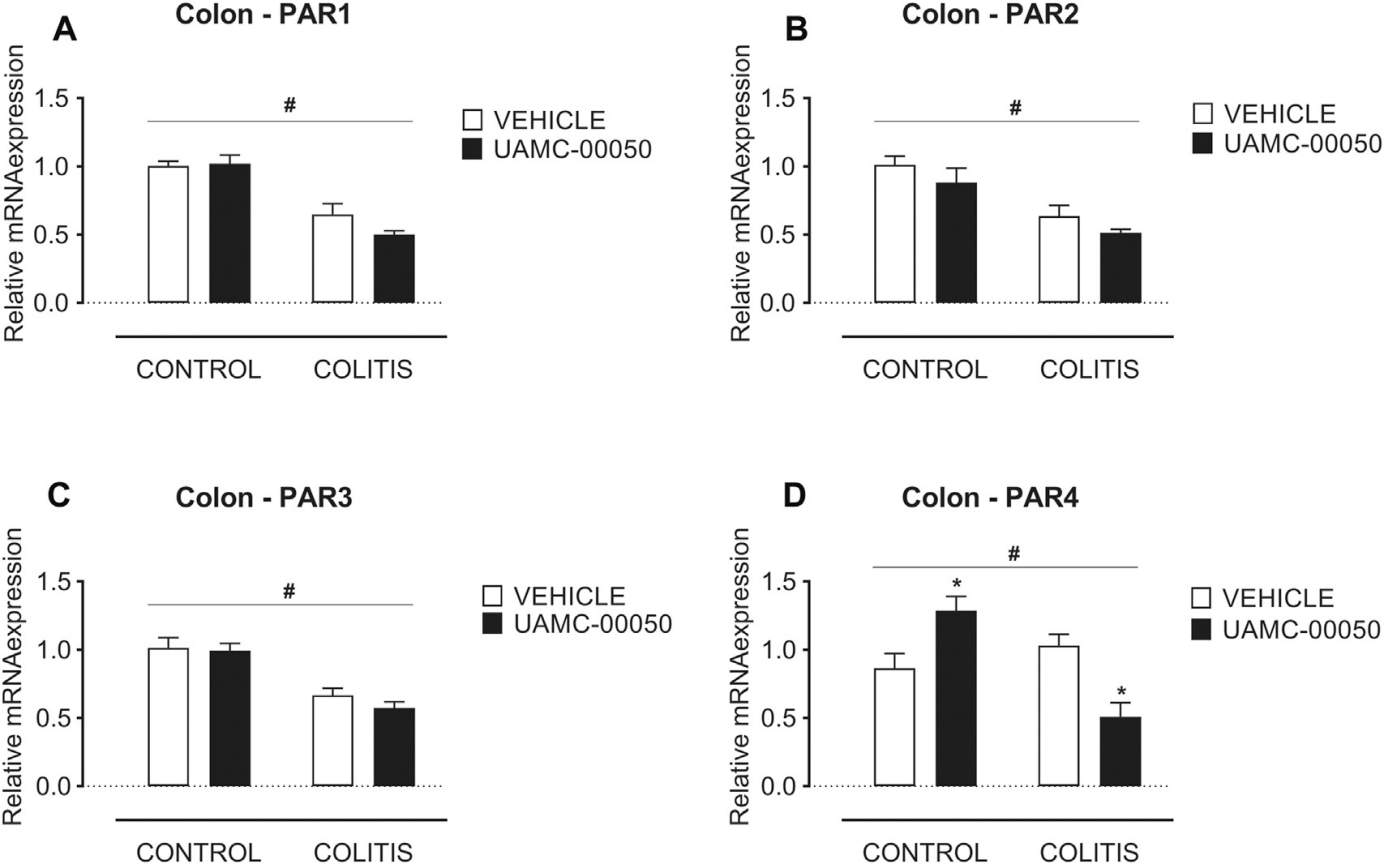
Effect of curative treatment with UAMC-00050 on colonic mRNA expression of protease-activated receptors. Effect on expression of PAR1 **(A)**, PAR2 **(B)**, PAR3 **(C)** and PAR4 **(D)**, significant effect of factor “colitis” and significant interaction between factor “colitis” and “treatment”. Data are determined post-mortem at week 4 (after a 2-week treatment with UAMC-00050). Data are represented as mean ± SEM. Two-way ANOVA followed by One-way ANOVA and LSD post-hoc test when appropriate. #*p* < 0.05; significant effect of colitis, *p < 0.05; significant effect of treatment, *n* = 7–8/group.

**FIGURE 6:**
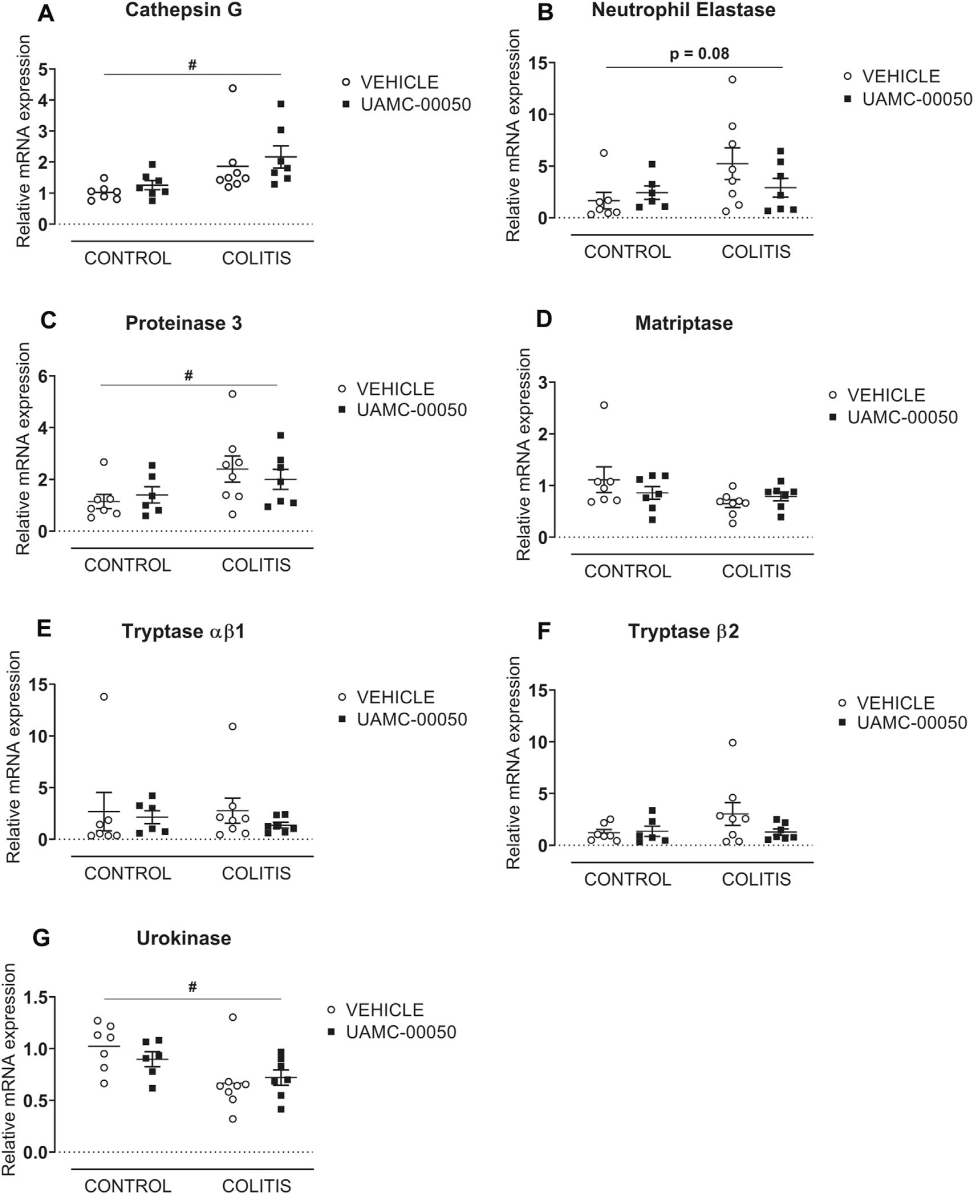
Effect of curative treatment with UAMC-00050 on colonic mRNA expression of serine proteases. Effect on expression of cathepsin G **(A)**, neutrophil elastase **(B)**, proteinase 3 **(C)**, matriptase **(D)**, tryptase αβ1 **(E)**, tryptase β2 **(F)** and urokinase **(G)**. Data are determined post-mortem at week 4 (after a 2-week treatment with UAMC-00050). Data are represented as mean ± SEM. Two-way ANOVA followed by One-way ANOVA and LSD post-hoc test when appropriate. #*p* < 0.05; significant effect of colitis, *n* = 7–8/group.

**FIGURE 7:**
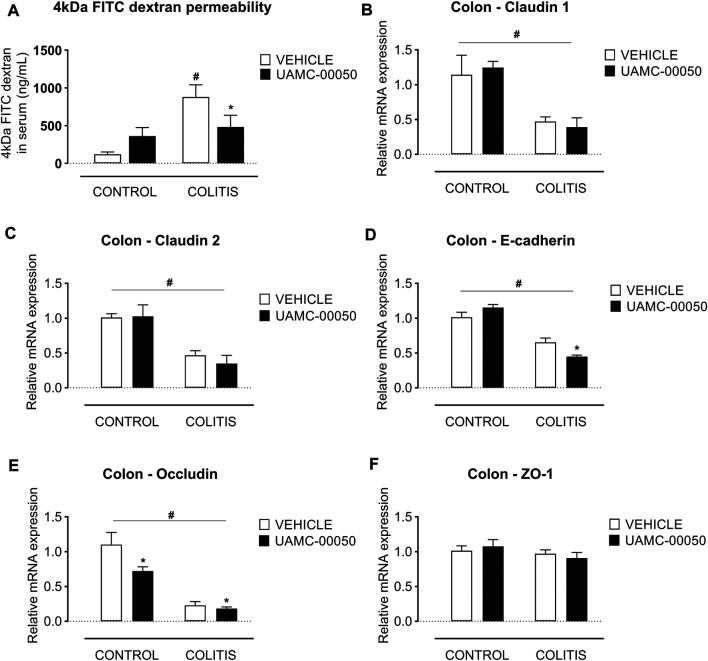
Effect of curative treatment with UAMC-00050 on intestinal permeability in experimental colitis. Effect on 4 kDa FITC-dextran intestinal permeability, significant interaction between factor “colitis” and “treatment” **(A)** and relative mRNA expression in the colon of the tight junctions claudin-1 **(B)**, claudin-2 **(C)**, E-cadherin **(D)**, occludin **(E)** and ZO-1 **(F)**. Data are determined post-mortem at week 4 (after a 2-week treatment with UAMC-00050). Data are represented as mean ± SEM. Two-way ANOVA followed by One-way ANOVA and LSD post-hoc test when appropriate. #*p* < 0.05; significant effect of colitis, *p < 0.05; significant effect of treatment, *n* = 7–8/group.

Adoptive transfer of CD4^+^CD25^−^CD62L^+^ T cells resulted in a significant and time-dependent weight loss and an increase in clinical disease score starting 2 weeks after transfer (Figures 2A,B). This was associated with the gradual development of colitis as evidenced by a significant increase in inflammatory indices assessed by colonoscopy, macroscopy, microscopy, MPO activity and colonic weight/length ratio (Figures 2C–G, Figure 3). Colitis also induced a significant increase in the colonic levels of the pro-inflammatory cytokines TNF-α, IFN-γ, IL-1β, IL-6 and IL-17 measured via CBA (Table 1). At the mRNA level, colitis induced a significant increase in Tbet and GATA-3, while the RORγt expression was significantly reduced (Figures 4A–C). Furthermore, the mRNA expression levels of PAR1, PAR2, PAR3 and PAR4 were significantly reduced by colitis (Figures 5A–D). The mRNA expression levels of the serine proteases cathepsin G, proteinase 3 and neutrophil elastase were upregulated, while matriptase and urokinase were downregulated and tryptase αβ1 and tryptase β2 were unaltered in colitis compared to control animals (Figures 6A–G). Finally, the intestinal permeability was investigated, showing an increased permeability for 4 kDa FITC-dextran in colitis mice (Figure 7A) associated with a decreased mRNA level of junctional proteins Cldn1, Cldn2, Cdh1 and Ocln (Figures 7B–E) and an unaltered expression of scaffolding protein Zo1 (Figure 7F).

**TABLE 1:** Effect of curative treatment with UAMC-00050 on cytokines in colon measured by CBA. Data are determined post-mortem at week 4 (after a 2-week treatment with UAMC-00050). Two-way ANOVA followed by One-way ANOVA and LSD post-hoc test when appropriate.

Cytokines	Control + vehicle	Control + UAMC-00050	Colitis + vehicle	Colitis + UAMC-00050
CBA colon (pg/ml)/100 mg tissue
TNF-α	3.8 ± 1.2	22.6 ± 3.5	176.4 ± 20.7^a^	74.7 ± 13.3^a^ ^,^ ^b^
IFN-γ	0.3 ± 0.2	4.8 ± 1.6	218.1 ± 29.4^a^	150.2 ± 39.5^a^
IL-1β	5.3 ± 1.2	17.6 ± 3.8	211.8 ± 34.5^a^	90.3 ± 24.0^a^ ^,^ ^b^
IL-2	1.8 ± 0.4	6.8 ± 0.9^b^	3.3 ± 0.2	2.8 ± 0.3
IL-6	0.9 ± 0.5	7.4 ± 1.1	24.6 ± 3.7^a^	10.4 ± 2.5^a^ ^,^ ^b^
IL-10	1.0 ± 0.5	26.8 ± 5.0^b^	0.7 ± 0.5	2.8 ± 1.2
IL-17	0.3 ± 0.1	3.5 ± 0.5^b^	4.2 ± 0.5^a^	2.4 ± 0.4^b^
IL-23	0.4 ± 0.2	6.9 ± 1.2^b^	1.8 ± 0.5	0.5 ± 0.2

a *p* < 0.05; significant effect of colitis.

b *p* < 0.05; significant effect of treatment, *n* = 7–8/group.

### Effect of UAMC-00050 on Inflammation and Intestinal Permeability Parameters During Experimental Colitis

Treatment with the serine protease inhibitor UAMC-00050, started at week 2 after adoptive T cell transfer, showed no significant effect on the clinical disease score in colitis mice (Figure 2B; Supplementary Table S2), whereas it moderately but significantly decreased the colonoscopic, macroscopic and microscopic score at week 4, associated with a significant decrease in the myeloperoxidase activity and a small but significant decrease in the colonic weight/length ratio (Figures 2C–G, Figure 3). Remarkably, treatment with UAMC-00050 significantly lowered the body weight in colitis mice at week 3 and in control mice at week 4 (Figure 2A). UAMC-00050 treatment also significantly decreased protein levels of TNF-α, IL-1β, IL-6 and IL-17 present in the colons of the colitis animals (Table 1). Remarkably, treatment with UAMC-00050 significantly increased the level of IL-2, IL-10, IL-17 and IL-23 in control animals (Table 1).

Besides, UAMC-00050 partially reversed the colitis-induced increase in Tbet and GATA-3 expression (Figures 4A,B) without affecting controls. On the contrary, UAMC-00050 treatment slightly but significantly increased RORγt expression (Figure 4C) both in controls and colitis animals.

UAMC-00050 treatment had no effect on the mRNA levels of PAR1, PAR2 and PAR3 (Figures 5A–C) in colitis or control mice, but altered the PAR4 mRNA expression with an increase in control animals and a decrease in colitis animals (Figure 5D).

Moreover, no significant differences in mRNA expression of different serine proteases were observed after treatment with UAMC-00050 compared to vehicle-treated colitis animals (Figures 6A–G).

Finally, treatment of mice with UAMC-00050 modestly but significantly ameliorated the colitis-induced impairment of intestinal permeability, measured by 4 kDa FITC-dextran (Figure 7A). Furthermore, UAMC-00050 fairly but significantly lowered the *Cdh1* expression level in colitis mice and the *Ocln* expression level in both control and colitis mice without having any effects on the mRNA level of any other junctional proteins in colitis or control mice (Figures 7B–F).

## Discussion

The intestinal mucosal barrier has a fundamental role in health and disease and disruption of this barrier has been put forward as a hallmark feature of various gastrointestinal diseases such as IBD and IBS (Van Spaendonk et al., 2017; Vancamelbeke and Vermeire, 2017). Moreover, the development of novel strategies to restore the intestinal barrier has been put forward to prevent CD in susceptible individuals, thereby highlighting the clinical relevance of this study (Turpin et al., 2020). The gastrointestinal tract is exposed to high levels of proteases and increasing evidence suggests that a dysregulation of the protease/antiprotease balance in the gut contributes to epithelial damage and increased intestinal permeability (Van Spaendonk et al., 2017). The aim of this study was to examine the effect of serine protease inhibition on inflammatory parameters and intestinal barrier function in the adoptive T cell transfer colitis mouse model which is characterized by intestinal inflammation and mucosal barrier dysfunction as recently described (Breugelmans et al., 2020).

Treatment with the newly developed selective serine protease inhibitor UAMC-00050 had a small but significant effect on colonic inflammation in T cell transfer colitis mice. This was reflected by an improvement of colonoscopic, macroscopic and microscopic scores, MPO activity and the colonic weight/length ratio. The decreased body weight seen in control and colitis animals after a 2-week treatment with UAMC-00050 could possibly be attributed to the long-term i.p. administration 2x/day, because side effects were not observed after an acute treatment scheme (Ceuleers et al., 2018). The beneficial effect of protease inhibitors on intestinal inflammation has also been reported previously in multiple colitis mouse models. More specifically, Motta *et al.* demonstrated that administration of the endogenous elastase inhibitor elafin, by transgenic expression or via adenoviral delivery, significantly improved intestinal inflammation in acute and chronic dextran sodium sulfate (DSS), trinitrobenzene sulfonic acid (TNBS) and chronic T cell transfer colitis mouse models (Motta et al., 2011; Motta et al., 2012). Recombinant acid lactic bacteria (recLAB) secreting serine protease inhibitors [elafin and secretory leukocyte protease inhibitor (SLPI)] induced more pronounced anti-inflammatory effects in a DSS-induced colitis mouse model compared to recLAB expressing anti-inflammatory cytokines (IL-10 and TGF-β1) (Bermudez-Humaran et al., 2015). Also soybean trypsin inhibitor-treated wild-type mice had reduced levels of *Citrobacter rodentium*-induced infectious colitis (Hansen et al., 2005), whereas treatment with the serine protease inhibitors nafamostat and camostat improved the inflammatory parameters in TNBS-induced colitis rats (Isozaki et al., 2006) and in two human patients with ulcerative colitis (Senda et al., 1993; Yoshida and Yoshikawa, 2008). Taken together, our own and previously published data clearly show that serine protease inhibitors have the ability to improve inflammation in different IBD animal models as well as in patients with ulcerative colitis.

To further substantiate the beneficial effect of the serine protease inhibitor UAMC-00050 on intestinal inflammation, we investigated which inflammatory pathways are involved. The importance of mucosal immune homeostasis and the key role of CD4^+^ T cells herein is well established. However, when this immune balance is disturbed, T cells can be the main drivers of IBD (Imam et al., 2018). In particular, T helper 1 (Th1) and Th17 cells have previously been identified as important players in the development of inflammation in the chronic T cell transfer colitis mouse model (Ogino et al., 2011; Heylen et al., 2014; Imam et al., 2018). Also in this study, it was shown that cytokines produced by Th1 (i.e., TNF-α, IFN-γ) and Th17 cells (i.e., IL-17) or promoting Th17 cell differentiation (IL-1β, IL-6) were significantly increased in T cell transfer colitis mice compared to control mice. Treatment with UAMC-00050 subsequently decreased expression of TNF-α, IL-1β, IL-6 and IL-17. In addition, UAMC-00050 increased the protein levels of IL-2, IL-10, IL-17 and IL-23 in control mice. This remarkable effect could possibly be explained by the fact that the administration of a protease inhibitor in control mice disturbs the protease/antiprotease balance, resulting in the activation of IL-2 and IL-23 and eventually the activation of IL-22 in order to maintain mucosal integrity (Bauché et al., 2020). Besides, IL-10 and IL-17 have been linked to epithelial proliferation and repair (Andrews et al., 2018). Furthermore, the transcription factors Tbet, GATA-3 and RORγt influence naïve T cell development towards Th1, Th2 and Th17/Treg cells, respectively (Zhu and Paul, 2010; Mickael et al., 2020). In this study, mRNA expression levels of Tbet and GATA-3 were significantly increased in T cell transfer colitis mice (colitis + vehicle group) which could be normalized after treatment with UAMC-00050 (colitis + UAMC-00050 group). RORγt mRNA expression was however decreased in T cell transfer colitis mice, but significantly increased upon UAMC-00050 treatment. This latter phenomenon may be due to the fact that nonpathogenic Th17 cells might lose RORγt expression during colitis, converting them into the pathogenic Th1-like Th17 cells as recently proposed (Mickael et al., 2020). Furthermore, RORγt is also present in Treg cells (Mickael et al., 2020), which thereby demonstrates the therapeutic effect of increased RORγt expression levels. The above findings suggest a role for Th1, Th17 and Treg cell subsets in the mechanism of action by which UAMC-00050 ameliorates intestinal inflammation.

The effect of serine protease inhibition on intestinal permeability was also investigated in the present study. It was previously demonstrated that the pro-inflammatory response and intestinal barrier dysfunction and disruption go hand in hand in the development of chronic colitis in the T cell transfer model, shown by an increased 4 kDa FITC-dextran permeability and an altered mRNA expression of the junctional proteins *Cldn1*, *Cldn2, Cdh1 and Ocln* (Breugelmans et al., 2020). Here, treatment with UAMC-00050 had a small but significant effect on the permeability towards 4 kDa FITC-dextran and also reduced the mRNA expression levels of *Cdh1* and *Ocln* in T cell transfer colitis mice. To our knowledge, we are the first to present the beneficial effect of serine protease inhibitors on intestinal barrier function in an *in vivo* IBD animal model. Earlier, Motta *et al.* reported that the protease inhibitor elafin caused a decreased FITC-dextran permeability in inflammation-induced Caco2-cells (Motta et al., 2011; Motta et al., 2012).

Taking a closer look at the possible mechanisms of action by which UAMC-00050 treatment ameliorated both intestinal inflammation and permeability, we first hypothesize a crosstalk between T cells and PARs as recently demonstrated for Treg cells and PAR4, where the activation of PAR4 on murine Tregs inhibits their function (Peng et al., 2019). In this study, the transcription factor RORγt, which can be expressed by Treg cells, marginally increased at mRNA level upon UAMC-00050 treatment in T cell transfer colitis mice, whereas PAR4 mRNA expression showed a modest but statistically significant decrease. This finding points towards a possible mechanism of action of UAMC-00050 on T cells, mediated via PAR4. However, further studies are needed to elucidate this hypothesis.

Moreover, the cross-talk between innate and adaptive immune cells on the one hand and the intestinal epithelium on the other hand is critical to maintain the mucosal barrier integrity and to avoid chronic inflammation and tissue damage (van der Gracht et al., 2016).

Another possible mechanism of action could be via a direct effect on the epithelium. Intestinal epithelial cells are an important source of proteases and they regulate the intestinal barrier permeability (Sola Tapias et al., 2020). For example, trypsin has been shown to induce pain and inflammation (Solà Tapias et al., 2017), while chymotrypsin, elastase and matriptase have been shown to increase intestinal permeability (Swystun et al., 2009; Buzza et al., 2010; Motta et al., 2012). Furthermore, PAR2 and PAR4 are expressed on epithelial surfaces (Mulè et al., 2004; Ceuleers et al., 2018) and the activation of PAR2 in intestinal epithelial cells by PAR2 agonists or by the serine proteases trypsin, tryptase and chymase induces inflammation and an increased colonic permeability in rodent models (Cenac et al., 2002; Coelho et al., 2002). In our study, we observed a possible role for cathepsin G, which was increased in colitis animals, and PAR4, which modestly but significantly decreased after treatment with UAMC-00050. Indeed, the serine protease cathepsin G and PAR4 have been demonstrated to play a role in inflammation and an increased colonic paracellular permeability (Dabek et al., 2009; Dabek et al., 2011).

The exact inhibitory profile against the different members of the serine protease family is of great importance. Denadai-Souza and colleagues demonstrated an overactive protease activity of thrombin and cathepsin G in the colonic mucosa of IBD patients compared to healthy controls (Denadai-Souza et al., 2018). Interestingly, we also observed an upregulation of cathepsin G at the mRNA level in this colitis mouse model. Moreover, UAMC-00050 has submicromolar IC_50_ values for several serine proteases, among which thrombin and cathepsin G (Ceuleers et al., 2018), thereby revealing a possibly interesting inhibitory profile for IBD patients. In this aspect, we want to highlight the importance of a further characterization of proteases and endogenous protease inhibitor levels in IBD patient cohorts and the design of highly specific inhibitors for defined subsets of patients (Sola Tapias et al., 2020). Moreover, synthetic small molecule serine protease inhibitors such as UAMC-00050 could also interfere with the interaction of the target serine proteases and endogenous serpins (Kriaa et al., 2020), which could then affect the clearing of proteases within the cell and thereby also the regulation of their expression patterns. After treatment with UAMC-00050, cathepsin G mRNA levels remained unaltered, but we want to emphasize that there could possibly be an effect of UAMC-00050 on other trypsin-like serine protease activity levels (Burster et al., 2010).

To conclude, we have investigated the effect of the serine protease inhibitor UAMC-00050 in a T cell transfer colitis mouse model. After a 2-week treatment, beneficial effects were observed with regard to the colonic inflammatory parameters as well as the intestinal barrier function. Furthermore, we hypothesize two different possible mechanisms of action (Chang, 2020): via the suppression of pro-inflammatory Th1 and activation of Treg cell differentiation on its turn possibly mediated through PAR4 signaling and/or (Mehandru and Colombel, 2020) via a direct effect on the epithelial barrier with a possible role for PAR4 resulting in fewer passage of luminal content, and subsequently less activation of the immune system (Th1 cells) and eventually less inflammation. More extensive studies are needed to unravel the therapeutic role of specific serine protease inhibitors on inflammation and intestinal barrier function as an additional or alternative therapy to the classic anti-inflammatory therapies for IBD patients.

## Data Availability

The raw data supporting the conclusions of this article will be made available by the authors, without undue reservation.
