# Supplementary material for: The Effect of a Novel Serine Protease Inhibitor on Inflammation and Intestinal Permeability in a Murine Colitis Transfer Model
Source: Front Pharmacol. 2021 Jun 24;12:682065. doi: 10.3389/fphar.2021.682065 (PMC8264366; doi:10.3389/fphar.2021.682065)
Supplement: Supplementary file 1 [file DataSheet1.docx]

Supplementary Material

# Supplementary Tables

# Supplementary table S1. Primers sequences used in qPCR assays.

| Species | Gene name | Primer | Primer sequence (5'-3') |
| --- | --- | --- | --- |
| Mouse | GAPDH | FW  REV | CCAGTATGACTCCACTCACG  GACTCCACGACATACTCAGC |
| Mouse | RPS29 | FW  REV | GTCTGATCCGCAAATACGGG  AGCCTATGTCCTTCGCGTACT |
| Mouse | RPL4 | FW  REV | CCGTCCCCTCATATCGGTGTA  GCATAGGGCTGTCTGTTGTTTTT |
| Mouse | Tbet | FW  REV | AGCAAGGACGGCGAATGTT  GGGTGGACATATAAGCGGTTC |
| Mouse | GATA3 | FW  REV | CTCGGCCATTCGTACATGGAA  GGATACCTCTGCACCGTAGC |
| Mouse | RORγT | FW  REV | GACCCACACCTCACAAATTGA  AGTAGGCCACATTACACTGCT |
| Mouse | PAR1 | FW  REV | TGAACCCCCGCTCATTCTTTC  CCAGCAGGACGCTTTCATTTTT |
| Mouse | PAR2 | FW  REV | GAAACACCCCGCCGTGATTTA  CTCCCCGTAGACCCAGTTG |
| Mouse | PAR3 | FW  REV | CCACCACAACTATAAAAGCGGA  GGTCACGATGTTGGCTGGTA |
| Mouse | PAR4 | FW  REV | CCAACGACAGTGACACGCT  GCCACCACAAGCCCATAGAG |
| Mouse | Ctsg | FW  REV | AGAAGACTTCGTCCTAACAGCA  CCTTTCTCGCATTTGGATGTTGT |
| Mouse | NeuElast | FW  REV | CAGGAACTTCGTCATGTCAGC  AGCAGTTGTGATGGGTCAAAG |
| Mouse | Proteinase 3 | FW  REV | CCCACTCTCGGCCTTATGTG  CGAATCTCGGGTGGATCAGG |
| Mouse | St14 | FW  REV | CTGGATGCGTATGAGAACTCC  TACAGCCGACTTCTTGTGGTA |
| Mouse | Tpsab1 | FW  REV | GCCAATGACACCTACTGGATG  GAGCTGTACTCTGACCTTGTTG |
| Mouse | Tpsb2 | FW  REV | CTGGCTAGTCTGGTGTACTCG  CCAGGGCCACTTACTCTCA |
| Mouse | Plau | FW  REV | GCGCCTTGGTGGTGAAAAAC  GACACGCATACACCTCCGTT |
| Mouse | Cldn1 | FW  REV | TGCCCCAGTGGAAGATTTACT  CTTTGCGAAACGCAGGACAT |
| Mouse | Cldn2 | FW  REV | CAACTGGTGGGCTACATCCTA  CCCTTGGAAAAGCCAACCG |
| Mouse | Cdh1 | FW  REV | CAGGTCTCCTCATGGCTTTGC  CTTCCGAAAAGAAGGCTGTCC |
| Mouse | Ocln | FW  REV | GGCGGATATACAGACCCAAGAG  GATAATCATGAACCCCAGGACAAT |
| Mouse | Zo-1 | FW  REV | GAGCGGGCTACCTTACTGAAC  GTCATCTCTTTCCGAGGCATTAG |

# Supplementary table S2. Clinical disease score – individual scores for different parameters

| Week | Clinical disease parameter | Control + vehicle | Control + UAMC-00050 | Colitis + vehicle | Colitis + UAMC-00050 |
| --- | --- | --- | --- | --- | --- |
| Week 1 | Weight loss | 1.29 ± 0.36 | 0.57 ± 0.20 | 1.13 ± 0.30 | 0.57 ± 0.20 |
|  | Pilo-erection | 0.07 ± 0.07 | 0.00 ± 0.00 | 0.06 ± 0.06 | 0.07 ± 0.07 |
|  | Mobility | 0.00 ± 0.00 | 0.00 ± 0.00 | 0.00 ± 0.00 | 0.14 ± 0.14 |
|  | Stool consistency | 0.14 ± 0.14 | 0.29 ± 0.18 | 0.13 ± 0.13 | 0.00 ± 0.00 |
|  | **Total score** | **1.50 ± 0.39** | **0.86 ± 0.14** | **1.31 ± 0.39** | **0.79 ± 0.21** |
| Week 2 | Weight loss | 0.43 ± 0.20 | 0.71 ± 0.36 | 1.00 ± 0.27 | 1.71 ± 0.29 |
|  | Pilo-erection | 0.00 ± 0.00 | 0.14 ± 0.14 | 1.31 ± 0.13 | 1.00 ± 0.11 |
|  | Mobility | 0.00 ± 0.00 | 0.00 ± 0.00 | 0.63 ± 0.18 | 0.50 ± 0.15 |
|  | Stool consistency | 0.14 ± 0.09 | 0.07 ± 0.07 | 1.00 ± 0.23 | 1.21 ± 0.18 |
|  | **Total score** | **0.57 ± 0.17** | **0.93 ± 0.38** | **3.94 ± 0.53** | **4.43 ± 0.54** |
| Week 3 | Weight loss | 0.14 ± 0.14 | 0.43 ± 0.20 | 1.38 ± 0.32 | 2.00 ± 0.00 |
|  | Pilo-erection | 0.00 ± 0.00 | 0.14 ± 0.14 | 1.63 ± 0.13 | 1.64 ± 0.09 |
|  | Mobility | 0.00 ± 0.00 | 0.00 ± 0.00 | 1.13 ± 0.23 | 1.21 ± 0.15 |
|  | Stool consistency | 0.14 ± 0.09 | 0.00 ± 0.00 | 1.44 ± 0.24 | 1.71 ± 0.15 |
|  | **Total score** | **0.29 ± 0.15** | **0.57 ± 0.30** | **5.56 ± 0.62** | **6.57 ± 0.13** |
| Week 4 | Weight loss | 1.00 ± 0.22 | 2.00 ± 0.00 | 2.00 ± 0.00 | 1.43 ± 0.30 |
|  | Pilo-erection | 0.00 ± 0.00 | 0.86 ± 0.09 | 1.75 ± 0.09 | 1.71 ± 0.10 |
|  | Mobility | 0.00 ± 0.00 | 0.43 ± 0.13 | 1.25 ± 0.13 | 1.64 ± 0.14 |
|  | Stool consistency | 0.00 ± 0.00 | 0.00 ± 0.00 | 1.81 ± 0.13 | 1.50 ± 0.15 |
|  | **Total score** | **1.00 ± 0.22** | **3.29 ± 0.18** | **6.81 ± 0.25** | **6.29 ± 0.31** |
